# Prevention of incisional surgical site infection by subfascial closed suction drainage after open laparotomy: a single surgeon experience in 250 consecutive patients

**DOI:** 10.1186/s13037-023-00354-z

**Published:** 2023-02-20

**Authors:** Hiroshi Isozaki

**Affiliations:** Department of Surgery, Oomoto Hospital, 1-1-5 Oomoto, Okayama, 700-0924 Japan

**Keywords:** Incisional surgical site infection, Laparotomy, Gastroenterological surgery, Subfascial closed suction drainage, Surgical site infection

## Abstract

**Background:**

Open laparotomy with gastroenterological surgery is a surgical procedure results in a relatively high rate (about 10% or more) of incisional surgical site infection (SSI). To reduce incisional SSI after open laparotomy, mechanical preventors, such as subcutaneous wound drainage or negative-pressure wound therapy (NPWT), have been tried; however, conclusive results have not been obtained. This study evaluated the prevention of incisional SSI by first subfascial closed suction drainage after open laparotomy.

**Methods:**

A total of 453 consecutive patients who underwent open laparotomy with gastroenterological surgery by one surgeon in one hospital (between August 1, 2011, and August 31, 2022) was investigated. Same absorbable threads and ring drapes were used in this period. Subfascial drainage was used in consecutive 250 patients in the later period (between January 1, 2016, and August 31, 2022). The incidences of SSIs in the subfascial drainage group were compared to those of in the no subfascial drainage group.

**Results:**

(a) No incisional SSI (superficial and deep) occurred in the subfascial drainage group (superficial = 0% [0/250] and deep = 0% [0/250]). As a result, incidences of incisional SSI of the subfascial drainage group were significantly lower than those of the no subfascial drainage group (superficial = 8.9% [18/203]; deep = 3.4% [7/203]) (*p* < 0.001 and *p* = 0.003, respectively). (b) Four out of seven deep incisional SSI patients in the no subfascial drainage group underwent debridement and re-suture under lumbar or general anesthesia. (c) There was no significant difference in the incidences of organ/space SSI of the two groups (3.4% [7/203] in the no subfascial drainage group and 5.2% [13/250] in the subfascial drainage group) (*P* = 0.491).

**Conclusion:**

Subfascial drainage was associated with no incisional SSI after open laparotomy with gastroenterological surgery.

## Introduction

Incisional surgical site infection (SSI) [1] is a troublesome postoperative complication. It is rarely fatal but leads to long-term hospitalization and physical and mental distress.

Open laparotomy with gastroenterological surgery is a surgical procedure that is performed with a clean-contaminated wound by wound classification [1, 2].　Thus, it often results in a relatively high rate (about 10% or more) of incisional SSI [3–5].

Numerous risk factors for developing an incisional SSI have been identified. Currently, to reduce incisional SSI after open laparotomy, mechanical preventors, such as subcutaneous wound drainage [6–10] or negative-pressure wound therapy (NPWT) [11–14], have been tried; however, conclusive results have not been obtained [15, 16].

Incisional SSI after laparotomy often occur after colorectal surgery or abdominal cavity contamination [3, 17, 18], and it is often accompanied by organ/space SSI [18, 19].

Under the hypothesis that incisional SSI might be prevented by shutout contaminated fluid raising from abdominal cavity, we started the subfascial closed suction drainage in all patient after open laparotomy from about halfway through this retrospective study period.

Herein, we report the first subfascial closed suction drainage to prevent incisional SSI after open laparotomy.

## Methods

For this retrospective cohort study, a total of 453 consecutive surgical patients who underwent open laparotomy (length of incision was 10 cm or more) by one surgeon in Oomoto hospital (between August 1, 2011, and August 31, 2022) was investigated. Patients who underwent laparoscopic surgery (*n* = 71 [6 stomach, 30 colorectum, and 35 gallbladder]) were excluded. The same absorbable threads, ring drape wound protector, and technical procedures of wound closure for fascia, subcutaneous tissue and skin, were used in this period.

Patients who died before the 30^th^ operative day were excluded from this study (*n* = 2; an 87-year-old gastric cancer patient who underwent fundectomy died of cardiopulmonary failure, and a 91-year-old advanced colonic cancer patient who underwent bypass operation died of uncontrollable bleeding from the tumor).

### Surgical technique

The surgeon performed all the surgical procedures from skin incision to skin closure. A skin incision was made by a scalpel, and subcutaneous fat, fascia, and peritoneum were separated with electrocautery. Wound protection during the operation was performed by ring drape.

Between August 1, 2011, and December 31, 2015, wound closure was performed by interrupted sutures using 1–0 Coated Vicryl*Plus (Antibacterial)® for the fascia together with peritoneum in 203 patients. After closure, the wound was irrigated with 500 ml of saline solution, and the subcutaneous fat tissue was closed by interrupted sutures using 3–0 Coated Vicryl®. Skin closure was made by continuous intradermal suture using 4–0 Monocryl®.

**Fig. 1:**
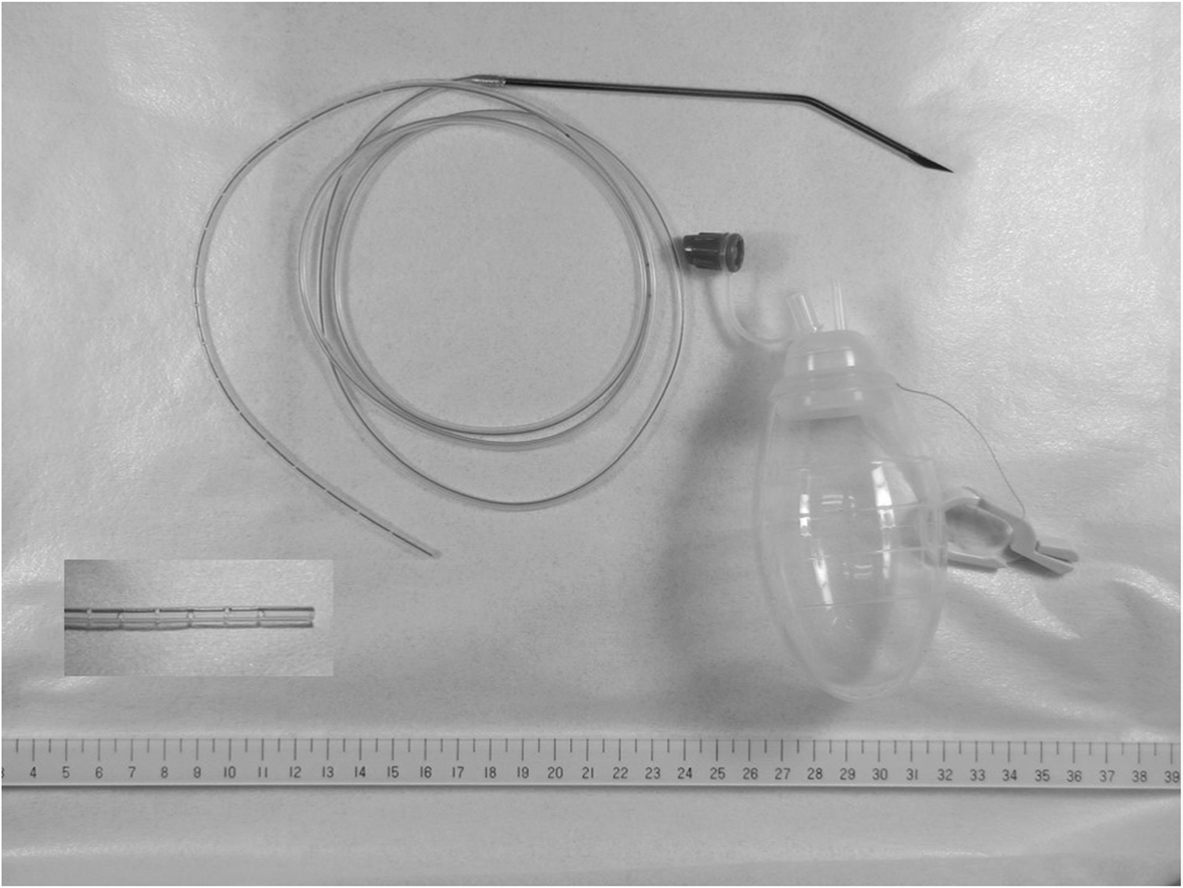
7F conventional drain tube with discontinuous small holes (tkb SurgicalProducts, TOKIBO)® and a low-pressure (30–80 mmHg), continuous-aspiration portable reservoir (Bulb-type 100 ml)

**Fig. 2:**
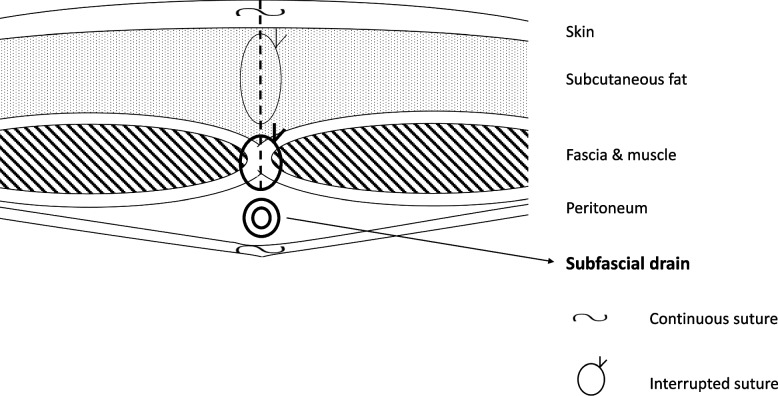
Schema of closure of abdominal wall and placement of subfascial drain tube

**Fig. 3:**
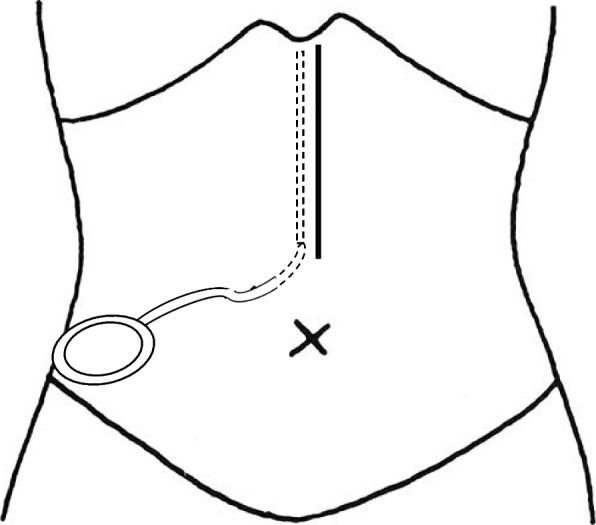
Subfascial drainage along the full length of the subfascial incision. Exit of the drain was placed separate from the incision at the caudal site, and connected to a low-pressure (30–80 mmHg), continuous-aspiration portable reservoir (Bulb-type 100 ml)

Between January 1, 2016, and August 31, 2022, wound closure was performed using the following procedure in 250 patients. Continuous suture of the peritoneum by 3–0 Coated Vicryl® was performed, and the wound was irrigated with 500 ml of saline solution. Then, a 7F conventional drain tube with discontinuous small holes (tkb SurgicalProducts, TOKIBO)® (Fig. 1) was placed between the peritoneum and fascia (under-muscle if the incision is subcostal or sub-umbilical) along the full length of the subfascial incision. The exit of the drain was placed separate from the incision at the caudal site. After this, the fascia was closed by interrupted sutures using 1–0 Coated Vicryl*Plus (Antibacterial)®. The same procedure as the no subfascial drainage group was performed for the closure of subcutaneous tissue and skin (Fig. 2). Finally, a drain tube was connected to a low-pressure (30–80 mmHg), continuous-aspiration portable reservoir (Bulb-type 100 ml) to allow the full length of the wound to be drained (Fig. 3). After the suturing of the wound, conventional gauze dressing was used in both groups.

### Outcome measures

The patient’s individual clinical items were recorded from their medical chart.

The following factors in relation to SSI were recorded: sex, age, body mass index, serum albumin, smoking, diabetes mellitus, previous laparotomy, emergency operation, blood transfusion, stoma-related, American Society of Anesthesiology score, organs of disease, malignancy, types of operations, sites of incision, wound classification, drainage from abdominal cavity, re-operation, and postoperative hospital stay. Hepatobiliary pancreatic diseases consisted of: liver cancer (*n* = 6), biliary tract cancer (*n* = 4), and pancreatic cancer (*n* = 4) in the no subfascial drain group; and liver cancer (*n* = 5), biliary tract cancer (*n* = 2), gallstones (*n* = 6), pancreatic cancer (*n* = 8), and pancreatitis (*n* = 1) in the subfascial drainage group.

The diagnosis of SSI was made by surgeons according to the criteria of the Center for Disease Control and Prevention (Table 1).

**Table 1:** Criteria of surgical site infection (summarized)

Surgical Site Infections (SSI)
**Incisional SSI**
**Superficial incisional SSI**
1. Purulent drainage, with or without laboratory conformation, from the superficial incision
2. Organisms isolated from an aseptically obtained culture of fluid or tissue from the superficial incision
3. At least one of the following signs or symptoms of infection: pain or tenderness, localized swelling, redness or heat, which requre the superficial incision to be deliberately opened by a surgeon,unless the incision is culture negative
4. Diagnosis of superficial incisional SSI made by the surgeon
**Deep incisional SSI**
1. Purulent drainage from the deep incision but not from the organ/space component of the surgical site
2. Diagnosis of a deep incisional SSI made by the surgeon
**Organ/space SSI**
1. Purulent drainage from a drain that is placed though a cut
2. Organisms isolated from an aseptically obtained culture of fluid or tissue in the organ/space
3. An abscess or other evidence of infection involving the organ/space that is found on direct examination, during reoperation, or by histopathologic or radiologic examination
4. Diagnosis of an organ/space SSI by the surgeon

SSIs were defined according to these definitions and occurring within 30 days after surgery

For the elective colorectal surgery, mechanical bowel preparation was performed two days before surgery. Preoperative oral antibiotics consisting of oral antibiotic mechanical bowel preparation were administered 2 days before surgery and the day of surgery.

Routine use of prophylactic antibiotics was as follows. For stomach disease, a first-generation cephalosporin (cefazoline sodium), and for the other diseases, a second-generation cephalosporin (flomoxef sodium) was administered by intravenous injection within 30 min before skin incision. In patients who underwent operations lasting longer than 3 h, additional doses of the same antibiotics were injected intravenously. These agents were also administered twice a day up to POD 3, according to the surgeon’s routine use.

All statistical analyses were performed using EZR (Saitama Medical Center, Jichi Medical University, Saitama, Japan), which is a graphical user interface for R (The R Foundation for Statistical Computing, Vienna, Austria) [20]. *P*-values < 0.05 by Fisher's exact test and unpaired *t*-test were considered significant.

## Results

The mean retention period of the subfascial drain was 5.9 days after surgery. Usually, the subfascial drain was removed simultaneously when the drain from the abdominal cavity was removed (mean = 6.0 days), and if no abdominal drain was inserted, the subfascial drain was removed (mean = 4.9 days) after surgery. The mean of the total volume of the subfascial drainage was 29.8 ml.

Table 2 shows the clinical items and incidences of the incisional SSI and the organ/space SSI in the no subfascial drainage group and the subfascial drainage group.

**Table 2:** Clinical items and incidences of the incisional SSI and the organ/space SSI in the no subfascial drainage group and the subfascial drainage group

Subfascial suction drainage				
		**No**	**Yes**	*p* value
Number of patients		**203**	**250**	
Sex	Male	115 (56.7%)	146 (58.4%)	0.774
	Female	88 (43.3%)	104 (41.6%)	
Age		68.84 (10.62)	69.77 (11.48)	0.374
Body mass index		21.80 (3.84)	22.44 (4.07)	0.089
Serum albumin (g/dl)		3.87 (0.52)	3.94 (0.49)	0.112
Smoking		37 (18.2%)	78 (31.2%)	0.002
Diabetes mellitus		10 (5.0)	15 (6.0)	0.684
Previous laparotomy		30 (14.8)	53 (21.2)	0.088
Emergency		10 (5.0)	32 (12.8)	0.005
Blood transfusion		45 (22.2)	49 (19.7)	0.561
Stoma-related		11 (5.4)	13 (5.2)	1
Organs of disease	Stomach	117 (57.6)	111 (44.4)	0.133
	Small Bowel	7 (3.4)	15 (6.0)	
	Colon	36 (17.7)	60 (24.0)	
	Rectum	26 (12.8)	37 (14.8)	
	Hepatobiliary pancreas	14 (6.9)	22 (8.8)	
	Others	3 (1.5)	5 (2.0)	
Malignancy		193 (95.1)	220 (88.0)	0.012
ASA score	1	88 (43.3)	89 (35.6)	0.103
	2	96 (47.3)	124 (49.6)	
	3	19 (9.4)	37 (14.8)	
Types of operations	Gastrectomy (partial)	79 (38.9)	81 (32.4)	NA
	Gastrectomy (total)	36 (17.7)	26 (10.4)	
	Colectomy	36 (17.7)	56 (22.4)	
	Rectal anterior resection	21 (10.3)	33 (13.2)	
	Miles or Hartmann	4 (2.0)	5 (2.0)	
	Total pelvic exenteration	1 (0.5)	1 (0.4)	
	Ileus (adhesiolysis)	3 (1.5)	3 (1.2)	
	Ileus (anastomosis)	3 (1.5)	9 (3.6)	
	Hepatobiliary pancreatic	14 (6.9)	22 (8.8)	
	Others	6 (3.0)	14 (5.6)	
Sites of Incision	1 Median (supra-umblical)	113 (55.7)	112 (44.8)	0.079
	2 Median (Median)	28 (13.8)	47 (18.8)	
	3 Median (sub-umbilical)	37 (18.2)	54 (21.6)	
	4 Subcostal	2 (1.0)	9 (3.6)	
	5 Subcostal + median	20 (9.9)	20 (8.0)	
	6 Right para-rectal	3 (1.5)	8 (3.2)	
Wound classification	2	202 (99.5)	243 (97.2)	0.08
	3	1 (0.5)	9 (3.6)	
Operative time (min)		135.8 (56.67)	165.5 (76.8)	< 0.001
Blood loss (ml)		138.1 (214.00)	143.7(221.7)	0.787
Drainage from abdominal cavity		161 (79.3)	214 (85.6)	0.081
Re-operation		6 (3.0)	11 (4.4)	0.467
**Incisional SSI**	**Superficial**	**18 (8.9)**	**0 (0.0)**	** < 0.001**
	**Deep**	**7 (3.4)**	**0 (0.0)**	**0.003**
Organ/space SSI		7 (3.4)	13 (5.2)	0.491
	Abscess	3 (42.9)	3 (23.1)	4 (30.8)
	Leakage	4 (57.1)	5 (38.5)	
	Bowel Perforation	0 (0.0)	1 (7.7)	
	Others	0 (0.0)	4 (30.8)	
Postoperative hospital stay (days)		26.8 (14.3)	24.52 (14.7)	0.093

*SSI *Surgical Site Infections

*ASA * American Society of Anesthesiology score

Comparing the two groups, the subfascial drainage group included more smokers and more emergency operations than the no subfascial drainage group. The rate of malignancy in the no subfascial drainage group was higher (95.1%) than that in the subfascial drainage group (88.0%). Moreover, the operative time (165.5 min) of the subfascial drainage group was longer than that of the no subfascial drainage group (135.8 min).

The incidences of re-operation were similar in the two groups (3.0% [6/203] in the no subfascial drainage group and 4.4% [11/250] in the subfascial drainage group) (*P* = 0.467).

### Surgical site infection

#### Incisional surgical site infection

A total of 18 incisional SSI was diagnosed at 10.5 postoperative days on average (5-18 days) in the no subfascial drainage. Diagnosis of incisional SSI was made by the surgeon himself in 5 patients and made by 5 other surgeons in 13 patients during routine doctor rounds. Then, the wounds were opened by those surgeons.

As a result of this study, no incisional SSI (superficial and deep) occurred in the subfascial drainage group (superficial 0% [0/250] and deep 0% [0/250]). Therefore, the incidences of incisional SSI of the subfascial drainage group were significantly lower than those of the no subfascial drainage group (superficial 8.9% [18/203] and deep 3.4% [7/203]) (*p* < 0.001 and *p* = 0.003).

In all seven deep incisional SSI patients in the no subfascial drainage group, superficial SSIs were found. Furthermore, four out of seven patients with deep SSI underwent debridement and re-suture under lumbar or general anesthesia. Re-suturing of the wound was performed by interrupted transdermal vertical mattress sutures with 2.0 monofilament nylon.

Bacterial test performed in ten patients with incisional SSI: Enterococcus faecalis (*n* = 2), Staphylococcus aureus (*n* = 1), Pseudomonas aeruginosa (*n* = 4) and Negative (*n* = 3).

### Organ/space surgical site infection

There was no significant difference in the incidences of organ/space SSI of the two groups (3.4% [7/203] in the no subfascial drainage group and 5.2% [13/250] in the subfascial drainage group) (*P* = 0.491). Four of 7 patients with organ/space SSI in the no subfascial drainage group was accompanied by incisional SSI. On the other hand, none of the 13 organ/space SSI patients in the subfascial drainage group was accompanied by incisional SSI (*p* = 0.007).

Table 3 shows incidences of incisional SSI according the organs of disease in the no subfascial drainage group and the subfascial drainage group. In colon or rectum group, the incidence of incisional SSIs was significantly different between the two groups.

**Table 3:** Incidences of the incisional SSI acccording to organs of disease in the no subfascial drainage group and the subfascial drainage group

Subfascial suction drainage					
	**No**		**Yes**		*p* value
Organs	Number of patients	Incisional SSI	Number of patients	Incisional SSI	
Stomach	117 (117)	5(5)	111(110)	0	0.06
Small Bowel	7(1)	1(1)	15(6)	0	0.318
Colon	36(34)	5(4)	60(49)	0	**0.007**
Rectum	26(26)	5(5)	37(36)	0	**0.009**
Hepatobiliary pancreas	14(13)	2(2)	22(15)	0	0.144
Others	3(2)	0	5(4)	0	1

*SSI *Surgical Site Infections

() Malignant disease

## Discussion

Various risk factors associated with SSI have been reported. Fukuda reported that intra-operative blood transfusion, diabetes, and use of steroids were risk factors for SSI following gastrointestinal surgery [21].

Although prophylactic antibiotics were administered up to POD 3 according to the surgeon’s routine use, in the period of the present study, we administered the following treatments according to the common recommendations: Oral antibiotic mechanical bowel preparation for elective colorectal surgery [22], a ring drape as a wound protector [3], an absorbable 1–0 Coated Vicryl*Plus (Antibacterial)® [23] for interrupted suture of the fascia [24], an absorbable 3–0 coated Vicryl for continuous suture of the peritoneum in the subfascial drainage group, an absorbable 3–0 coated Vicryl for interrupted suture of subcutaneous fat mass, and an absorbable 4–0 Monocryl® for continuous intradermal suture of skin closure [25].

As mentioned before, open laparotomy with gastroenterological surgery is an operation performed with a clean-contaminated wound, resulting in relative high rate (over 10%) of incisional SSI.

In the present study, our hypothesis was that incisional SSI might be prevented by shutout of contaminated fluid raising from the abdominal cavity; however, there has been no report of subfascial closed suction drainage to prevent incisional SSI after open laparotomy. On the other hand, there have been many reports concerning subcutaneous drainage after laparotomy to prevent incisional SSI; however, conclusive results have not been obtained.

In a previous review of subcutaneous drainage, the advantage of closed suction drainage over passive drainage was not shown [15]. Moreover, among studies of subcutaneous closed suction drainage, the results varied [8, 15]. In a review of subcutaneous wound drainage in reducing surgical infection after laparotomy, Manzoor et al. [15] stated that: “There seems to be no benefit in using it in clean and clean contaminated wounds. However, there may be benefit in using drains in patients who are at high risk, including patients who are obese and/or have contaminated wound types.”　A recent systematic review and meta-analysis demonstrated that the use of subcutaneous suction drains did not exhibit any significant differences between drained and undrained patients in developing SSI (odds ratio 0.76, 95% CI 0.56–1.02; *p* = 0.07) [26].

NPWT as a mechanical preventor has been tried, as well as open dirty wound, to reduce incisional SSIs of closed incisions after laparotomy. NPWT has several possible mechanisms, including the prompt removal of exudation to avoid fluid on the inter-stitched face and tissue layers [13]. However, similar to subcutaneous closed suction drainage, conclusive results have not been obtained [14, 16]. Recently, a systematic review and meta-analysis of randomized trials of prophylactic NPWT for closed laparotomy wounds showed that the overall SSI rate in NPWT groups (18.6%, 87/467) was significantly lower than that of standard dressing groups (23.9%, 111/464) (Odds ratio 0.71, 95% CI 0.52–0.99, *p* = 0.04*) [27].

Subcutaneous suction drainage and NPWT were performed under the hypothesis that the elimination of dead space and fluid collection by active suctioning may prevent wound infection. However, our findings suggest that the beneficial effect of only subcutaneous suctioning remains to be shown.

In the present study, subfascial drainage resulted in no incisional SSIs (0/250) after open laparotomy with gastroenterological surgery. In comparison to the no subfascial drainage group, the subfascial drainage group consisted of more smokers, more previous laparotomies, more emergency operations, worse wound classification, and longer operative time.

Now, we could not understand the preventing mechanism of incisional SSI by the subfascial suction drainage which yielded this striking result. However, the following mechanism might be caused: contaminated fluid from the abdominal cavity sucked below the fascial space; in addition, the exudate under the subcutaneous space sucked through the gap between sutures of the facia.

This retrospective cohort study of incisional SSI has the following limitations. First, this study was performed by one surgeon in one hospital; thus, it was not a randomized trial. Second, the period of study could be divided into early (no subfascial drainage) and late (with the subfascial drainage) periods. The main shortcomings related to historic controls is the introduction of "hidden bias" related the multiple additional standards in care. Although the same ring drape for wound protection and same absorbable threads for the closure of abdominal wall were used in both periods of this study, some changes of infection prevention protocols or gastrointestinal surgical procedures may have occurred. Third, this study may be the first report of the subfascial suction drainage after laparotomy; thus, randomized controlled trials are necessary to confirm the present findings.

## Conclusion

In conclusion, subfascial drainage was associated with no incisional SSI (0/250) after open laparotomy with gastroenterological surgery. Based on the insights from this study, we recommend the placement of the subfascial suction drainage after open laparotomy, especially after colorectal surgery or abdominal cavity contamination, to prevent incisional SSI.

## Data Availability

The datasets used and/or analyzed in this study are available from the corresponding author upon reasonable request.

## Abbreviations

SSI: Surgical site infection
NPWT: Negative-pressure wound therapy
